# Economic Cost Analysis of West Nile Virus Outbreak, Sacramento County, California, USA, 2005

**DOI:** 10.3201/eid1603.090667

**Published:** 2010-03

**Authors:** Loren M. Barber, Jerome J. Schleier, Robert K.D. Peterson

**Affiliations:** Montana State University, Bozeman, Montana, USA

**Keywords:** West Nile virus, Sacramento County, California, mosquito vector control, economic cost analysis, vector-borne infections, viruses, research

## Abstract

In 2005, an outbreak of West Nile virus (WNV) disease occurred in Sacramento County, California; 163 human cases were reported. In response to WNV surveillance indicating increased WNV activity, the Sacramento-Yolo Mosquito and Vector Control District conducted an emergency aerial spray. We determined the economic impact of the outbreak, including the vector control event and the medical cost to treat WNV disease. WNV disease in Sacramento County cost ≈$2.28 million for medical treatment and patients’ productivity loss for both West Nile fever and West Nile neuroinvasive disease. Vector control cost ≈$701,790, including spray procedures and overtime hours. The total economic impact of WNV was $2.98 million. A cost-benefit analysis indicated that only 15 cases of West Nile neuroinvasive disease would need to be prevented to make the emergency spray cost-effective.

## Abstract 2

Aerial spraying is cost-effective.

After its introduction into the eastern United States in 1999, West Nile virus (WNV) reached California in 2003 (*1*). In response, the state enhanced mosquito management programs to reduce vector populations and virus transmission (*2*). By late summer 2005, WNV disease was epidemic in Sacramento County, with more cases reported in Sacramento County than in any other county in the nation that year (*3*). The Sacramento-Yolo Mosquito and Vector Control District (SYMVCD) responded by conducting emergency aerial spraying over the city of Sacramento and surrounding areas to reduce mosquito populations.

Effective management of infection rates, illness, and death from mosquito-borne pathogens such as WNV requires reduced contact between humans and infected mosquitoes (*4*). No effective treatment exists for WNV; prevention of disease relies on management of mosquitoes through various control tactics. Elnaiem et al. (*5*) and Carney et al. (*6*) examined the efficacy of the 2005 emergency aerial spray in Sacramento County, which used pyrethrins as the active ingredients to control adult mosquitoes. In both studies, an unsprayed area within the county was used as the control. Elnaiem et al. showed a total decrease in WNV-competent vector mosquitoes, *Culex pipiens* and *Cx. tarsalis*, of 57.5%, compared with the prespray population in the treated area (*5*). They also observed a decrease in WNV infection rates in mosquitoes to 3.9/1,000 for trapped females in the treated areas, compared with 6.7/1,000 in the untreated areas (*5*). Carney et al. used illness onset dates and residential locations for 152 of the 163 WNV disease cases reported in humans in 2005 to determine the efficacy of the spray event (*6*). Their results showed no incident human cases in the treated area after the spray event, compared with 18 cases in the untreated area. Consequently, the emergency aerial spray seemed to effectively reduce both mosquito populations (*5*) and human WNV cases (*6*).

WNV infection can be asymptomatic or symptomatic in humans, with a 4:1 ratio (*7**,**8*). The disease can be mild, resulting in influenza-like symptoms (as in West Nile fever [WNF]), or severe, affecting the central nervous system symptom (as in West Nile neuroinvasive disease [WNND]) (*7*). Many WNF cases are not reported because they are not recognized as WNF; symptoms can resemble a cold or mild influenza-like illness, for which medical care is not sought, or is underdiagnosed because the additional cost of testing would not provide alternative direction to effective palliative medical care (*7**,**9*).

Zohrabian et al. (*10*) estimated the economic impact of the WNV disease outbreak in 2002 in Louisiana, which resulted in 24 deaths. They included costs of inpatient and outpatient medical care, productivity loss, the state’s public health department, and vector control. Total epidemic costs were ≈$20.14 million for the 329 cases, including $9.2 million for mosquito control and public health agency costs. Zohrabian et al. (*11*) used the economic data from their 2004 study to determine the cost-effectiveness of the initiation of a potential WNV vaccination and found that the cost of vaccination would not offset the costs in medical care.

Several studies have demonstrated the efficacy of mosquito management in response to WNV, but only the study by Carney et al. (*6*) suggested a reduction in human WNV cases associated with aerial adult-mosquito control. We estimated the economic cost of the 2005 WNV disease outbreak in Sacramento County, California, and evaluated the reduction in WNV disease necessary to offset the cost of emergency vector control. Economic costs for patients’ productivity loss and for treatment of disease symptoms, as well as for emergency vector control conducted in response to the outbreak were also investigated.

## Methods

### Medical Costs

We estimated costs for the total number of Sacramento County WNV cases in 2005. Different costs were associated with WNF and the more severe WNND. The Centers for Disease Control and Prevention (CDC) summarizes the reported number of WNV cases for each state, including patient’s age, sex, date of onset, case reporting date, county of residence, diagnosis (WNF or WNND), and outcome (e.g., fatal). According to the CDC database for 2005, a total of 935 human WNV cases were reported in California, including 163 cases from Sacramento County (*3*). A total of 117 (71.8%) were diagnosed as WNF and 46 (28.2%) as WNND; 1 (0.6%) case was fatal. Forty-six (28.2%) patients were >60 years of age, and 2 (1.2%) were <18 years of age.

For WNND, we calculated costs using similar methods as and specific data from Zohrabian et al. (*10*). Costs of inpatient and outpatient care, lost productivity, and miscellaneous expenses were summed to estimate the total cost of an individual WNND case. Costs for WNF, including average price for a physician visit, CDC-approved diagnostic testing, and productivity loss during symptomatic WNV disease, were summed to estimate the total cost of an individual WNF case.

#### WNND

We obtained inpatient costs for WNND using the 2005 hospital patient discharge database from California’s Office of Statewide Health Planning and Development (OSHPD) (J. Teague and J. Morgan, pers. comm.). This database included patients with a WNV-related diagnosis who were admitted to hospitals within Sacramento County’s ZIP codes. It also included average inpatient hospital charge per stay and average length of stay for the different WNV diagnosis codes (Table 1). Cost data were available for 16 of the 27 WNND cases reported by Sacramento County hospitals in 2005 (some hospitals do not report cost data). Charges were averaged for each diagnosis code, and the average charge was determined for WNND (no hospital cases were reported for WNF). The average charge was then converted to the true economic cost by using the average Sacramento County hospital cost-to-charge ratio (CCR). Individual hospitals’ CCRs were obtained from California’s Department of Industrial Regulations (*12*), and the average was based on the number of cases reported at each hospital in the county, also obtained from OSHPD. The resulting inpatient cost was extrapolated to all WNND cases in Sacramento County for the total economic impact.

**Table 1:** WNF diagnosis codes and cases, Sacramento County, California, 2005*

Diagnosis code	Diagnosis	No. hospitalized case-patients reported†	Classification	Cases with cost data‡	Average ALOS§ (range¶)
66.40	WNF, unspecified	0	WNF	0	
66.41	WNF with encephalitis	17	Severe (WNND)	14	16 (12–36)
66.42	WNF with other neurologic manifestation	8	Severe (WNND)	2	13 (10–14)
66.49	WNF with other complications	2	Severe (WNND)	0	21 (13–29)
WNND totals or averages		27		16	15 (10–36)

*WNF, West Nile fever; ALOS, average length of stay (J. Teague and J. Morgan, pers. comm.); WNND, West Nile neuroinvasive disease. †Cases in 2005 obtained from the Patient Discharge Data from California’s Office of Statewide Health Planning and Development (J. Teague and J. Morgan, pers. comm.). ‡Cases that included cost data from the Patient Discharge Database and incorporated into the study. §Average length of stay, in days (J. Teague and J. Morgan, pers. comm.). ¶Not the true ALOS range, determined from data for each diagnosis code.

We estimated outpatient costs for WNND using the 2002 outpatient costs determined by Zohrabian et al. (*10*) and updated to 2005 using data from the Consumer Price Index (CPI) for the western United States, obtained from the US Department of Labor, Bureau of Labor Statistics (*13*–*15*). Zohrabian et al. used hospital cost data reported from 119 patients and phone surveys of 139 patients to determine related treatment costs for WNV disease symptoms. CPI data included the percentage increase for medical care services for 2002–2003, 2003–2004, and 2004–2005. These increases were applied to the service categories originated by Zohrabian et al.: hospital treatment, physician visits, outpatient physical therapy, occupational therapy, and speech therapy. Percentages of total patients for whom the service applied were determined by using information from Zohrabian et al. for each outpatient category; these percentages were then applied to the Sacramento County WWND cases for the costs per patient per category.

Miscellaneous costs included nursing home, transportation, home-health aides, and child care costs accrued during recovery from WNND. Average nursing home costs per day in 2005 were obtained from the Survey of Nursing Home and Home Care Costs (*16*). We calculated the value by averaging the national costs for the daily rate of a private and semiprivate room in 2005. The total associated costs for a nursing home stay was then determined by multiplying this value by the average number of days a WNND patient spent in a nursing home (96 days) (*10*). We applied this cost to 3.6% of the WNND patients and rounded it to the nearest whole number of patients. Transportation, home-health aides, child care, and other home-help costs were calculated by using the cost values determined in Zohrabian et al., updated to 2005 by using the CPIs mentioned previously (*13*–*15*). We applied the resulting transportation cost to all WNND cases, and applied costs for home-help aides to 14.4% of the 2005 WNND cases and rounded to the nearest whole number of patients.

We assumed that productivity loss differentially affected persons in 2 age groups: >60 years and <60 years. Productivity loss was also calculated for nonprofessional caretakers of WNND patients. We determined the cost for a day of work missed by an average Sacramento adult citizen using the mean annual earnings for full-time workers in 2005 (*17*). Annual income was divided by 250 work days per year. The resulting value was the cost for a day of work missed by persons <60 years of age. We calculated the cost for a nonwork day missed using Productivity Loss Tables from 2000 (*18*) and updated to 2005 dollars using the US Department of Labor, Bureau of Labor Statistics, annual earnings (*17*). The percentage increase from 2000 to 2005 was applied to the Productivity Loss Tables’ value for a nonwork day loss. The resulting value for a nonwork day missed also was used for productivity loss for persons >60 years of age who had WNND. We conservatively assumed an average of 50 work days missed (*10*) and 10 nonwork days missed (1 weekend day per week).Thus, total productivity loss was 60 days. For caretakers of WNND patients, productivity loss was assumed to be 25 days, and the associated cost was the value of a nonwork day missed (*10*). The cost attributed to productivity loss is an estimate; true monetary value for pain and distress and the productivity loss associated with chronic WNND are uncertain.

#### WNF

Assumed costs for treating WNF were those of a physician visit, a diagnostic test, and productivity loss during symptomatic WNF. We obtained the average costs for a physician visit for a diagnosis or treatment in the western United States from 2004 data (*19*) and updated to 2005, using the CPI (*15*) as discussed above.

The CDC-approved diagnostic test for human WNV is an immunoglobulin (Ig)M and IgG ELISA for either serum or cerebrospinal fluid (*7*). According to CDC, an additional test is needed to indicate a false-positive result; however, our analysis assumed only costs for the initial diagnostic test. We obtained this value by contacting 4 laboratories suggested by the California Department of Public Health (C. Jean, pers. comm.) (ARUP Laboratories, Salt Lake City, UT, USA; Focus Diagnostics Inc., Cypress, CA, USA; Quest Diagnostics Inc., Madison, NJ, USA; and Specialty Laboratories, Valencia, CA, USA); the costs obtained were then averaged. Productivity loss for a missed day of work and a missed day of nonwork were calculated by using the methods detailed previously. We assumed 5 workdays missed because of WNF for persons <60 years of age and 5 nonwork days missed for persons >60 years of age.

### Cost of Mosquito Vector Control

We obtained cost information for the 2005 emergency mosquito control aerial spray from SYMVCD. It included aerial ultra-low–volume adulticiding over 2 areas in Sacramento County comprising ≈477 km^2^ (*6*). Aerial spraying was conducted on 6 nights in early and mid-August (*5*). The event costs incorporated overtime hours for SYMVCD employees for August 2005. We calculated total overtime hours spent on the emergency spray using the difference between paid overtime hours for August 2005 and August 2004. Overtime hours for August 2005 were assumed to be additional hours to SYMVCD’s usual vector control program, including hours for additional prespray and postspray application mosquito trapping, plane preparation time, and preparation time for completing the spraying. These hours included time spent on other spray events and vector control procedures not directly involved in the emergency spray. However, our study incorporated total overtime hours for August to ensure conservatism. Total cost for the emergency spray also included outsource contracts (e.g., plane rental, pilot hours) and the insecticide used.

## Results

### Medical Costs for WNND

A total of 46 WNND cases occurred in Sacramento County in 2005. Costs were ≈$33,143 per inpatient and ≈$6,317 per outpatient for all treatments (Table 2). Cost for each WNND patient estimated to have spent time in a nursing home was ≈$18,097. Productivity loss during symptomatic WNND cost $10,800 per patient <60 years of age and $7,500 per patient >60 years of age (Table 3). Total medical costs accrued by all WNND patients was ≈$2,140,409; total costs for all cases (medical cost plus productivity loss) was ≈$2,844,338.

**Table 2:** Estimated inpatient and outpatient economic costs of WNND cases, Sacramento County, California, 2005*

Item	Cost per case†	No. cases to which cost applies‡	% Cases to which cost applies§	Total cost for all cases	Total cost if treatment/service were used in all cases
Inpatient treatment costs	$33,143	46	100	$1,524,570	$1,524,570
Outpatient costs	Cost per case¶				
Outpatient hospital treatment	$333	17	36	$5,668	$15,337
Physician visits	$450	46	100	$20,708	$20,708
Outpatient physical therapy	$909	46	100	$41,810	$41,810
Occupational therapy	$4,037	3	7	$12,111	$185,699
Speech therapy	$588	1	1	$588	$27,032
Total				$80,885	$290,586
Nursing home costs	Cost#				
Nursing home stay**	$190	2	4	$36,195	$36,195
Transportation	$65	46	100	$2,977	$2,977
Home health aides, babysitters, etc.	$1,569	7	14	$10,983	$505,211
Total				$50,154	$544,383
Total for WNND				$2,140,409	$2,844,339

*WNND, West Nile neuroinvasive disease; BLS, Bureau of Labor Statistics of the US Department of Labor. †Estimated by using 2005 data from California’s Office of Statewide Health Planning and Development (J. Teague and J. Morgan, pers. comm.). ‡WNND cases from the total number of cases reported by the Centers for Disease Control and Prevention (*3*). §See (*10*). ¶Estimated by using data from Zohrabian et al. (*10*) and updated using data from the US Department of Labor’s Bureau of Labor Statistics (BLS) (*13*–*15*). #Estimated by using data from MetLife Mature Market Institute (*16*), Zohrabian et al. (*10*), and BLS (*13*–*15*). **Average length of nursing home stay was 96 days.

**Table 3:** Estimated economic costs of WNND cases due to productivity loss, Sacramento County, California, 2005*

Productivity loss	Value of work day missed†	Value of nonwork day missed‡	No. work days missed	No. nonwork days missed	No. patients		% Cases	Total costs for all cases
					<60	>60		
For patients <60 y	$191	$125	50	10	31		100	$334,800
For patients >60 y		$125		60		15	100	$112,500
For caretakers		$125	25		8	4	26	$37,500
Total costs								$484,800

*WNND, West Nile neuroinvasive disease. †Estimated by using data from BLS (*17*). ‡Estimated by using data from Grosse (*18*) and BLS (*17*).

We performed sensitivity analysis for medical treatment of WNND in which we had a range of values using 10,000 iterations. The hospitals’ CCRs contributed the largest amount of variance to the total cost (68.5%), followed by the average inpatient cost per WNND patient from the 2005 hospital patient discharge database from OSHPD (J. Teague and J. Morgan, pers. comm.) (31.4%), range $1,910,421–$7,770,354. Results were similar for the cost per WNND inpatient (range $13,201–$140,257) and the total medical cost for treating WNND.

### Medical Costs for WNF

A total of 117 WNF cases were reported for Sacramento County in 2005. Treating each WNF patient cost ≈$167 for the diagnostic physician visit and ≈$135 for the diagnostic test. Productivity loss cost ≈$955 for each patient <60 years of age and $625 for each patient >60 years of age. The total cost for treating reported WNF cases was ≈$136,839 (Table 4).

**Table 4:** Estimated economic impact for WNF cases (N = 117), Sacramento County, California, 2005*

Item	Cost		No. patients by age, y		Total cost
			<60	>60	
Physician visit for diagnosis or treatment in the western US, cost per case†	$167		86	31	$19,539
Diagnostic tests, average cost per case‡	$135				
Productivity loss	Per work day missed§	Per nonwork day missed¶	Total individual cost#		
Value of a lost day	$191	$125	$955	$625	$101,505
Total costs for WNF					$136,839

*WNF, West Nile fever; BLS, Bureau of Labor Statistics, US Department of Labor. †Estimated by using data from Brown and Beauregard (*19*) and BLS (*15*). ‡ELISA immunoglobulin (Ig) G and IgM serum and cerebrospinal fluid. Estimated by using laboratory list prices (ARUP Laboratories, Salt Lake City, UT, USA; Focus Diagnostics Inc., Cypress, CA, USA; Quest Diagnostics Inc., Madison, NJ, USA; Specialty Laboratories, Valencia, CA, USA). §Estimated by using data from BLS (*17*). ¶Estimated by using data from Grosse (*18*) and BLS (*17*). #Based on 5 workdays missed per person <60 y and 5 nonwork days missed per person >60 y.

Sensitivity analysis for the cost of treating WNF (range $132,008–$144,458) showed that the average cost for the diagnosis test contributed the largest amount of variance to the total cost (84.2%). The cost of a missed day of work for patients <60 years of age was 15.8%.

### Emergency Vector Control Spray

The emergency spray comprised 1,157 additional overtime hours in SYMVCD for August 2005. These overtime hours cost ≈$41,790. The emergency spray cost ≈$660,000 (D. Brown, pers. comm.). Therefore, the emergency aerial spray response to the WNV epidemic cost a total of $701,790.

### Total Costs and Potential Benefits

Total cost of the 2005 Sacramento County WNV epidemic was ≈$2,979,037. Costs for treating WNND patients alone exceeded costs of emergency vector control by $1,438,619, a ratio of 3:1. This difference suggests that for the benefits of the vector control to outweigh the cost of the epidemic, the spray event would need to prevent only 15 WNND cases.

## Discussion

Since 1999, when WNV was detected in the United States, several studies have evaluated the efficacy of vector control, especially adulticide treatments. Palmisano et al. (*20*) observed an 86% decrease (compared with a 5-year average) in WNV-vectoring mosquitoes in 2002 resulting from control efforts over a 4-month period in St. Tammany Parish, Louisiana. Simpson (*21*) observed a 64% reduction in WNV-carrying mosquito species measured during emergency aerial sprays in 26 Florida counties during 2004 in response to hurricanes. Carney et al. (*6*) and Elnaiem et al. (*5*) provided evidence of the effectiveness of the 2005 emergency aerial spray as a mosquito control measure in Sacramento County by showing a reduction both in mosquito populations and WNV disease cases in humans.

Carney et al. (*6*) documented 18 total WNV disease cases outside the spray area after the Sacramento County emergency spray and no cases within the spray area, after they adjusted for the maximum incubation period of the virus from infection to onset of symptoms. Of these 18 cases, 13 were diagnosed as WNF and 5 as WNND. Treating these 18 patients cost ≈$241,462. However, given the possibility of unreported or underdiagnosed WNF cases, the spray event may actually have prevented >18 cases (*7**,**9*,*22*,*23*). SYMVCD activities conducted before the emergency period most likely prevented some cases.

Estimating the medical costs of WNV patients and the true number of cases prevented by the emergency spray are uncertain. The estimated dollar amount designated for productivity loss from WNV disease was based on the average annual salary of a Sacramento County citizen in 2005 and an estimated number of work days missed because of the disease. This study does not take into account extreme cases of WNND and total number of days a patient is affected by the disease. Therefore, the actual cost values associated with WNV may be higher.

Our analysis may underestimate the actual cost of the WNV outbreak. Pain and distress are difficult to estimate monetarily but probably are important factors in the comprehensive costs of WNV disease. We also did not include medical costs associated with non-WNV issues, such as mosquito-bite allergenicity or sequelae, which are difficult to quantify but may be substantial (*24*). Additionally, we did not incorporate the benefits to the human population of reducing the nuisance of mosquito bites, irrespective of WNV transmission. In Jefferson County, Texas, the ratio of the cost of the total household benefit to the program cost for mosquito abatement was 1.8, according to a countywide study on the benefit of mosquito control in reducing the nuisance of mosquito bites (*25*). In addition, the actual number of persons affected with WNF remains unknown because the total number of WNF cases probably was underreported and underdiagnosed (*7**,**9*). Busch et al. (*26*) found 353 infections for each reported case of WNND in North Dakota from blood screening data in 2003 compared with CDC data indicating ≈256 WNV incident infections for each WNND case in the United States.

We did not assess human and ecologic risks associated with the emergency spray. However, previous risk assessments that used exposure scenarios for pyrethroids and pyrethrins that would exceed those of the Sacramento County emergency aerial spray have shown risks substantially below Environmental Protection Agency levels of concern (*27*–*34*).

The total economic impact of the 2005 WNV disease outbreak in Sacramento County was ≈$2.98 million. The total cost of medical treatment for the outbreak was $2.28 million. The actual number of WNV disease cases prevented by the emergency spray is uncertain. However, the offset in cost for the number of cases that may have been prevented can be compared with the costs of the vector control. If only 34 WNF and 14 WNND cases (by using the percentages of each from the diagnoses for Sacramento County in 2005) were prevented by the spray event, ≈$702,809 would have been averted in medical and productivity loss costs, thus offsetting the cost of the emergency spray. Also, the costs of the emergency spray would have been offset by preventing only 15 WNND cases at ≈$706,833.
